# Development of a brain wave model based on the quantitative analysis of EEG and EEG biofeedback therapy in patients with panic attacks during the COVID-19 pandemic

**DOI:** 10.1038/s41598-022-19068-w

**Published:** 2022-09-01

**Authors:** Marta Kopańska, Danuta Ochojska, Wiktoria Mytych, Marcin W. Lis, Agnieszka Banaś-Ząbczyk

**Affiliations:** 1 Department of Pathophysiology, Institute of Medical Sciences, Medical College of Rzeszow University, Rzeszow, Poland; 2 Department of Psychology, Institute of Pedagogy, College of Social Sciences, University of Rzeszow, 35-959 Rzeszow, Poland; 3 Students Science Club “Reh-Tech”, University of Rzeszow, Rzeszow, Poland; 4 Department of Zoology and Animal Welfare, Faculty of Animal Science, University of Agriculture in Cracow, 30-059 Cracow, Poland; 5 Department of Biology, Institute of Medical Sciences, Medical College of Rzeszow University, Rzeszow, Poland

**Keywords:** Psychology, Environmental sciences, Health care

## Abstract

The current global crisis facing the world is the COVID-19 pandemic. Infection from the SARS-CoV-2 virus leads to serious health complications and even death. As it turns out, COVID-19 not only physically assails the health of those infected, but also leads to serious mental illness regardless of the presence of the disease. Social isolation, fear, concern for oneself and one's loved ones, all of this occurs when a pandemic overloads people. People exhibit numerous neurological disorders that have never happened to them before. Patients are diagnosed with frequent panic attacks, the result of which can be seen in their Quantitative Electroencephalogram results. This test may be one of the main diagnostic tools of the COVID-19 pandemic. From the results obtained, it is possible to compare and draw conclusions. This method of testing effectively allows EEG biofeedback training and observes its effect on brain activity. The feedback received in this way gives us the opportunity to properly tailor a protocol for the patient and their conditions. Numerous studies support the effectiveness of EEG biofeedback for panic attacks and other psychiatric disorders. The purpose of our study was to show the effectiveness of EEG biofeedback with a Quantitative Electroencephalogram of the brainwave pattern after having COVID-19 and what symptoms may result.

## Introduction

Announced on March 11, 2020 by the World Health Organization, the global COVID-19 pandemic caused by the SARS-CoV-2 virus has become a major threat to human health worldwide^1^. Typical symptoms of the disease are fever, cough, and shortness of breath. The course of the disease can lead to complications, including pneumonia and acute respiratory distress syndrome. Patients also report many long-term effects after infection, affecting multiple organs and sometimes entire systems in the body, including the nervous system^1–6^. The pandemic outbreak itself has not only affected infected people, but has also significantly affected the lives of the general population. Research clearly shows that the pandemic itself has had a negative impact on the mental health of many people^7–9^. Global events such as climate change, economic crises, or armed conflicts produce similar results, causing a mental burden for the human population. These events are associated with severe stress, affecting mental health and, in turn, the entire body. Environmental stress caused by fear of the pandemic, war, or other global crises promotes mental pathologies which lead to severe depressive disorders, obsessive–compulsive disorders, post-traumatic stress disorder, anxiety disorders, panic attacks, sleep disorders and similar non-psychotic disorders^10–13^. Numerous studies suggest that every global crisis goes hand in hand with a psychiatric epidemic. Thus, COVID-19 carries implications for both mental and physical health and currently, there is heightened anxiety and fear in society. In China, according to a study, searches for terms related to panic attacks and anxiety on the Internet increased significantly with the announcement of the COVID-19 pandemic^9^. Panic attacks, and by extension panic disorder characterized by cardiovascular, respiratory, gastrointestinal, and autonomic nervous system symptoms, became a major concern for the human population during the COVID-19 pandemic^14,15^. DSM-5 criteria place panic disorder in the class of anxiety disorders, characterized by excessive alertness and activity, which lead to significant changes in the emotional as well as cognitive and behavioral domains. There are many common symptoms that accompany anxiety disorders. The main ones are sweating, trembling, accelerated heart rate, heat strokes, hyperventilation, fear and many more. These symptoms can lead patients to a state of detachment from reality and even into psychosis^16–18^. A QEEG or an electroencephalogram is a popular and non-invasive method used in diagnostic brain imaging. The QEEG test allows the brain wave frequencies to be analyzed and compared to those typical of a patient with a panic disorder. Correct analysis of the results obtained from QEEG makes it possible to plan appropriate EEG biofeedback therapy^6,19–24^. According to Banerjee et al. the most common therapy for panic disorders, which also has increasingly better results, is biofeedback^25^. EEG biofeedback is a method widely used in various fields to control physiological processes in the body. Usually EEG biofeedback training is applied according to the principles of frequency—intensity—time—type (FITT). The training can be safely adapted to the patient to have the desired effects on the physical and mental zones. Psychophysical coherence provides a balance between both spheres^25–27^. This therapy provides us with feedback on the changes that occur in brain activity, allowing us to make adjustments to parameters after each workout if necessary. Thanks to this it makes it possible to influence and modulate in a controlled way the electrical activity of the brain. In our study we wanted to test the effectiveness of EEG biofeedback training based on QEEG imaging. We hypothesized a positive effect of the training on brain wave activity.

## Results

### Delta waves

**Figure 1:**
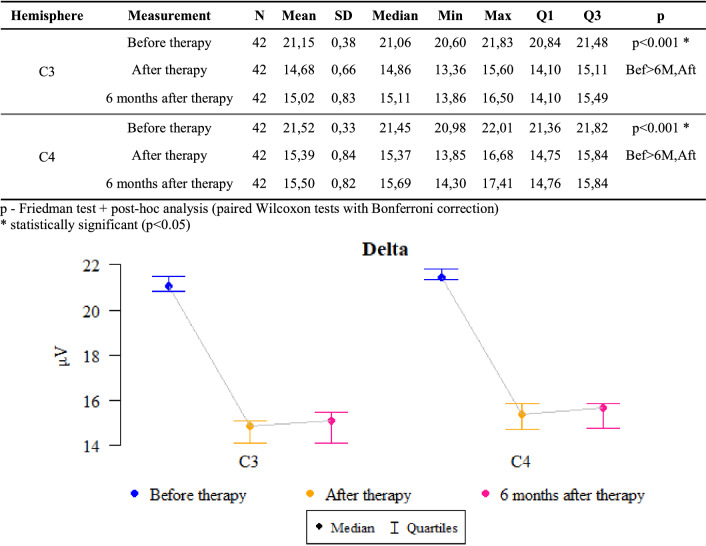
The results of the Delta waves examination.

*P* values ​​ < 0.05 indicate statistically significant dependences:

The amplitude at C3 and C4 points was significantly higher before the therapy than after it and after 6 months of follow-up. That is, the amplitude significantly decreased after the therapy as compared to the state before the therapy and this decrease was also maintained after 6 months (Fig. 1).

### Theta waves

*P* values < 0.05 indicate statistically significant dependences:

The amplitude at point C3 was significantly higher before the therapy than after it and after 6 months of follow-up. That is, the amplitude significantly decreased after the therapy as compared to the state before the therapy and this decrease was also maintained after 6 months.

**Figure 2:**
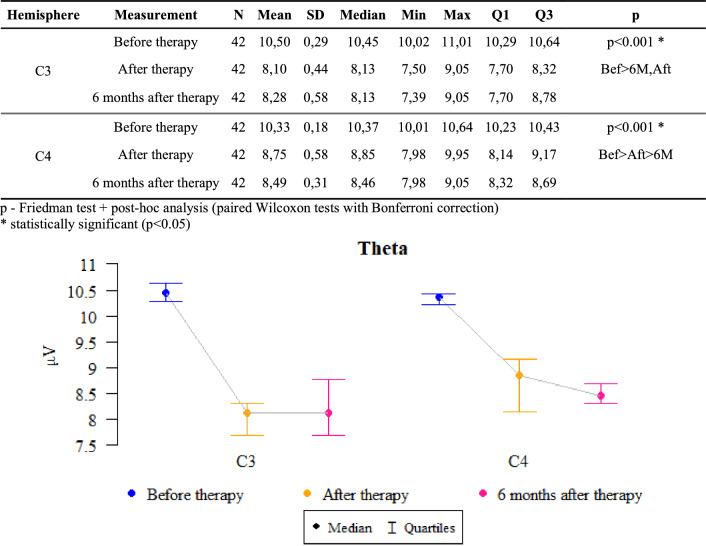
The results of the Theta waves examination.

The amplitude at the C4 point was significantly higher before the therapy than after it, when it was significantly higher than after 6 months. That is, the amplitude dropped significantly after the therapy compared to the state before the therapy, and this decrease even intensified after 6 months (Fig. 2).

### Alfa waves

**Figure 3:**
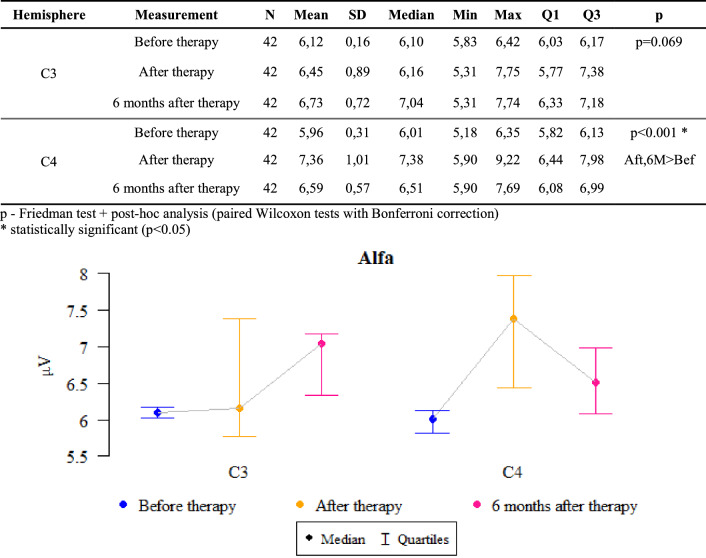
The results of the Alpha waves examination.

*P* values ​​ < 0.05 indicate statistically significant dependencies:

The amplitude at point C4 was significantly higher after treatment and after 6 months of follow-up than before treatment. That is, the amplitude increased significantly after therapy compared to the state before therapy and this increase was also maintained after 6 months (Fig. 3).

### SMR waves (sensorimotor waves)

*P* values ​​ < 0.05 indicate statistically significant dependencies:

The amplitude at C3 point was significantly higher before the therapy than after it and after 6 months. That is, the amplitude significantly decreased after the therapy as compared to the state before the therapy and this decrease was also maintained after 6 months.

**Figure 4:**
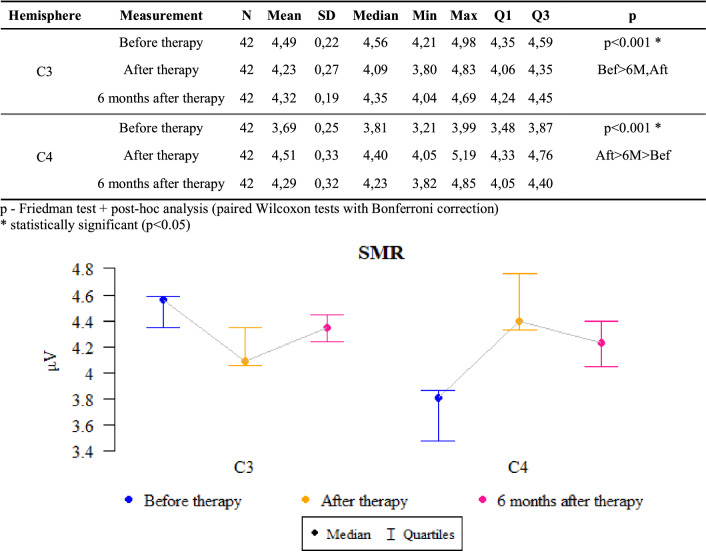
The results of the SMR waves examination.

The amplitude at point C4 was significantly higher after treatment than after 6 months, when it was significantly higher than before treatment. That is, the amplitude increased significantly after the therapy as compared to the state before the therapy, and then, after 6 months of follow-up, it dropped significantly, but still remained significantly higher than before the therapy (Fig. 4).

### Beta 1 waves

*P* values ​​ < 0.05 indicate statistically significant dependencies:

The amplitude at C3 point was significantly higher before the therapy than after it and after 6 months of follow-up. That is, the amplitude significantly decreased after the therapy as compared to the state before the therapy and this decrease was also maintained after 6 months.

**Figure 5:**
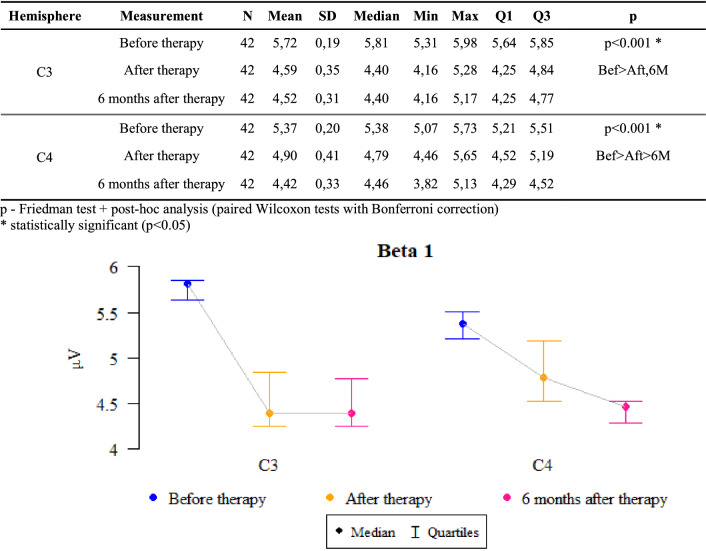
The results of the Beta1 waves examination.

The amplitude at C4 point was significantly higher before the therapy than after it, when it was significantly higher than after 6 months. That is, the amplitude dropped significantly after the therapy compared to the state before the therapy, and this decrease even intensified after 6 months (Fig. 5).

### Beta 2 waves

*P* values ​​ < 0.05 indicate statistically significant dependencies:

The amplitude at point C3 was significantly higher before the therapy than after it and after 6 months of follow-up. That is, the amplitude significantly decreased after the therapy as compared to the state before the therapy and this decrease was also maintained after 6 months.

**Figure 6:**
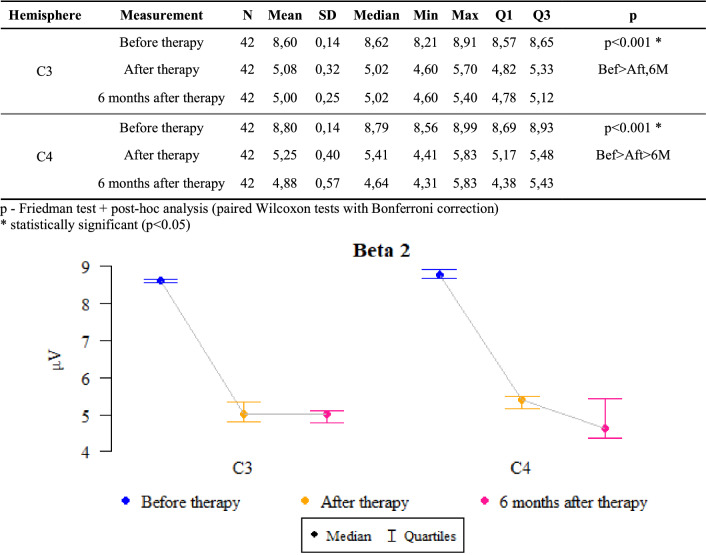
The results of the Beta2 waves examination.

The amplitude at the C4 point was significantly higher before the therapy than after it, when it was significantly higher than after 6 months. That is, the amplitude dropped significantly after the therapy compared to the state before the therapy, and this decrease even deepened after 6 months (Fig. 6).

## Discussion

Throughout world history, humanity has endured many pandemics, epidemics, disasters, and global catastrophes. Numerous publications have presented their consequences on the physical and mental health of populations. Currently, the COVID-19 pandemic has made its mark around the world. Andrea Fiorillo et al. in their work showed an increase in mental problems in patients with the outbreak of the COVID-19 pandemic^28^. Humans are social beings, seeking and needing contact with others. The mean of the IES-R analysis questionnaires conducted in China during the COVID-19 pandemic was 24 points, revealing the effects of PTSD^9^. Post-traumatic stress disorder (PTSD) is one of the most serious mental disorders caused by experiencing traumatic situations. Extreme feelings of danger and fear are triggered in the body such that people begin to experience anxiety and stress in normal situations^28–30^. Isolation, and thus the lack of physical contact and the ability to talk face to face, without the use of electronic devices and social distance have a detrimental effect on the psyche. Increased stress levels, significantly reduced interpersonal contacts and a tough economic situation significantly increase stress levels which affects the whole body, lowering the quality of life. The world situation has led people to extreme terror and consequently to serious mental disorders. Hossain MM. et al. found that the scale and significance of COVID-19 affects people’s mental condition, resulting in depression, panic, stress, anxiety, and sleep and somatic disorders^13^. There are also digestive, circulatory, immune, and mental problems. In addition, people infected with SARS-CoV-2 generally had higher levels of stress than those not infected. There is speculation that SARS-CoV-2 infection, affecting the central and peripheral nervous systems, contributes to a labile autonomic response with symptoms characteristic of panic disorder^31,32^. Psychological well-being is also highly dependent on the level of psychosocial stress. For healthcare workers, who are the most vulnerable to illness, this pandemic has become a test of mental toughness and a huge worry for their families. Other factors that affect the level of burden include social status, marital status, age, gender, occupation and simply the individual's psychological resilience. Place of residence and income during the pandemic also contributed greatly to mental health conditions^13^. Generally, people living in rural areas in isolation felt the effects of social distance much less than those living in urban areas. Adaptation and acceptance of changes in the surrounding environment is associated with resilience to symptoms of mental disorders. Nevertheless, Costanza et al. found in their study that adaptive coping strategies and levels of resilience did not significantly protect against the symptoms of stress, etc^33^. QEEG studies show a correlation between psychiatric disorders and amplitude change in brain waves. Pasini E. et al. in their study show the sensitivity of EEG testing in relation to post-COVID encephalopathies. The authors highlight the effectiveness of EEG testing in imaging complications after SARS-CoV-2 virus infection^34^. All of the patients we considered in our study had a history of complaints of tension, disorganization, anxiety, facial muscle twitching, problems with fluent speech, and problems with an unstable pulse. They sought additional forms of help after surviving COVID, including neurological and cardiac help. The neurologist recommended that they try undergoing EEG Biofeedback training tailored to their current brainwave status, based on a previously conducted QEEG diagnostic test. Two recurring phenomena in particular were observed in all patients: the first was an increase in mainly 2 types of waves: Delta and Theta, relative to controls. We primarily observed an increase in the amplitudes of these waves in the left hemisphere relative to the right hemisphere, which may possibly indicate a rather large hemispheric conflict, possibly affecting the synchronization of the peripheral sympathetic-parasympathetic system, which is likely to express itself in problems with judgment, social interactions and increased panic. Increased Beta 2 waves in both hemispheres were also noted, indicating intracranial tension. Beta 2 waves are often associated with increased feelings of anxiety, emotional tension and stress. These waves are also often accompanied by the release of adrenaline, which is responsible for mobilizing the body. Staying in this state of mind for too long can lead to excessive feelings of fatigue^35^, which was the case with the patients we analyzed. Keith J. Kincaid et al. in their paper present a patient after SARS-CoV-2 virus infection with neurological abnormalities of unknown origin, as seen in CT, MRI and EEG findings. At the time of symptom onset, the patient was negative for COVID-19, so the authors suggest "Long-COVID" neurological symptoms^36^. Marcele Regine de Carvalho et al. in their study showed a correlation between the presence of a panic disorder and agoraphobia and the variation in Beta wave activity. They found that the study group had an absolutely higher Beta wave in the frontal cortex compared to the control group^37^. This may be indicative of anxiety-based overstimulation. Our study also confirmed the correlation of increased Beta waves in individuals with panic attacks compared to the control group. These authors, in another paper, also found a state of high arousal in people with panic disorder and agoraphobia, showing high Alpha waves. As it turned out, the Alpha wave ratio in the study group is much higher than in the control group^37,38^. Pąchalska et al. showed that quantitative brainwave analysis (QEEG), like an EEG, has high sensitivity in terms of temporal resolution and is imaged in real time^39^. Thompson et al. reported that in their study of patients. Similar to the SARS-CoV-2 virus infection group presented in this article, with marked symptoms of emotional disturbance, a qualitative EEG was assessed as normal or at the limits of normal. Only quantitative analysis was able to better assess the current state of the brain and pinpoint the problem. With the QEEG technique, it is possible to determine the basis of abnormalities in the functioning of areas of the cerebral cortex and correlate the clinical condition with images of power maps and QEEG graphs^40^. The analyses of the QEEG results of five studied people with a generalized anxiety disorder^41^ show specific pattern amplitudes at C3 and C4. In all the patients, two dependencies were repeated: low contribution of SMR wave amplitudes and high Beta 2 wave amplitudes, higher or equal to the Alpha amplitudes. Therefore, the results of studies of people with panic attacks indicate that Beta 2 waves are mainly associated with severe anxiety, dominant in both generalized anxiety disorder and in panic disorder, which is an anxiety disorder. Going through such a pandemic and other global crises leads to a variety of problems on both physical and psychological grounds, but it has been observed that the results obtained after neurofeedback training are promising^42^. Patients themselves began to see the results of the training over time. Symptoms of mental disorders decreased significantly or disappeared completely. EEG biofeedback training has been shown to automatically have a positive impact on a patient's attitude and quality of life^43^. Hamwey, M. K. et al. in their study conducted on a group of 610 people focused on the effects of the traumatic events that took place on September 11, 2001. The terrorist attacks that took place at that time left a mark on the psyche of Americans, who had increased anxiety and depressive reactions. The mere memory of this period causes anxiety in many people^44^. Therefore, recovery and appropriate therapy can be crucial. Thanks to the performance of QEEG and the individualized selection of the EEG Biofeedback therapeutic protocol, satisfactory results were achieved in terms of improving emotional disorders in post-Covid patients. Moreover, after repeated QEEG diagnostic examination 6 months after the end of EEG Biofeedback therapy, the ranges of wave amplitudes remained at a similar level as immediately after therapy, and the patients' condition could be described as good. In interviews, the patients were calmer, the previously visible facial muscle twitching had passed, and speech was more fluent and better-organized. Orendáčová M. and Kvašňák E. also suggest neurofeedback as a key tool in the treatment of neurological complications after COVID-19^45^. By conducting multiple studies and critically comparing the effects of different factors, we can create a psychosocial epidemiology of the COVID-19 pandemic. This can be used to create a treatment protocol for psychiatric disorders.

## Limitations

Our study is not free from limitations. The main objection to the credibility of our work may become the rather small number of patients subjected to QEEG (42 patients). However, the people included had to show a diagnosed history of panic attacks, which first appeared in their lives during the COVID-19 pandemic (never before pandemic) and their negative impact on daily life of these people. Study design in order to narrow the scope of the study, we deliberately delimited. The study was designed for people from one geographical location. In doing so, it avoided significant ethnic diversity and climate change, which could suggest a limitation of the research results. An unintended consequence during data collection was that respondents signed up to participate (self selection bias). The limitations of the study research is the validity of its results. It refers to accuracy, reliability of the results and in general validity to the generalizability of the results from our study’s sample to the population. In our study due to internal validity we induced effects of events and changes in participants due to time. In order to mitigate limitations, patients in our study groups with different educational backgrounds, working in different occupations were included as part of the study design. However, we left the criterion of work activity in a given age range to equivalently avoid too much variance when generalizing the results. In addition, it was important in our study to be able to assess QEEG in the same patients after SARS-CoV-2 infection, before and after the EEG Biofeedback training course. This became one of the main limitations of the study, especially having the results directly after SARS-CoV-2 infection, before EEG Biofeedback. But it also created the uniqueness of the study and its main advantage.

## Conclusion

The QEEG study provides a link between the COVID-19 pandemic and post-infection neurological disorders. Patients with symptoms after SARS-CoV-2 virus infection can participate in QEEG-based EEG biofeedback training. EEG biofeedback training is one of the primary methods of treating mental disorders in people affected by strong stressors, including pandemics and other global crises. An EEG can be a useful tool to monitor changes of psychotherapy and pharmacological therapy, too. In the QEEG study, we observe abnormalities in brainwave frequencies. EEG biofeedback training potentially influences patients to return to their pre-pandemic mental state, which has a positive effect on the return to full mental function. The EEG biofeedback method helps to reduce mood disorders among patients. Cognitive and executive functions are improved and anxiety, panic, and fear are reduced. Our article opens the way to uncovering further mysteries in the brain function of post-COVID-19 patients manifesting symptoms of panic attacks. However, further research is needed, in particular, conducting a thorough QEEG analysis in all lobes of the cerebral cortex. In particular, the centers responsible for cognitive functions should be taken into account.

## Material and methods

### Patients

The study included 42 patients. In their medical histories, none of the patients declared any current or past psychiatric treatment. All patients were also found to have no neurological or psychiatric diseases in their family history. No comorbidities were present and no medications were taken during the study (Table 1).

**Table 1:** The table with basic information about patients.

	Sex	Age	Education	Employment
Patient 1	Male	41	Master’s degree	Personal trainer
Patient 2	Female	42	Secondary education	Teacher
Patient 3	Male	38	College education	Driver
Patient 4	Female	36	Master’s degree	Accountant
Patient 5	Female	36	Master’s degree	Physiotherapist
Patient 6	Female	41	Master’s degree	Banker
Patient 7	Male	39	Master’s degree	Electrician
Patient 8	Male	37	PhD	Lawyer
Patient 9	Female	35	Master’s degree	Stewardess
Patient 10	Male	36	Master’s degree	Teacher
Patient 11	Female	38	College education	Builder
Patient 12	Male	38	Master’s degree	Accountant
Patient 13	Female	40	College education	Driver
Patient 14	Male	37	College education	Entrepreneur
Patient 15	Female	35	PhD	lawyer
Patient 16	Female	42	PhD	doctor
Patient 17	Female	36	College education	Engineer
Patient 18	Male	35	PhD	Paramedic
Patient 19	Male	41	College education	Developer
Patient 20	Female	40	Master’s degree	Architect
Patient 21	Male	40	College education	Personal trainer
Patient 22	Male	42	Bachelor's degree	Truck driver
Patient 23	Female	37	Bachelor's degree	Office clerk
Patient 24	Female	41	Bachelor's degree	Teacher
Patient 25	Male	40	PhD	Construction worker
Patient 26	Female	37	PhD	Nurse
Patient 27	Male	36	College education	Web developer
Patient 28	Male	35	Bachelor's degree	E-commerce
Patient 29	Female	42	Master’s degree	Cashier
Patient 30	Female	40	Secondary education	Retailer
Patient 31	Female	41	PhD	Web developer
Patient 32	Male	39	Bachelor's degree	Cook chef
Patient 33	Female	42	PhD	Software engineer
Patient 34	Male	38	Master’s degree	E-commerce
Patient 35	Male	37	Master’s degree	Office clerk
Patient 36	Male	35	Master’s degree	Administrative assistant
Patient 37	Female	42	PhD	Biologist
Patient 38	Female	41	Master’s degree	Nurse
Patient 39	Male	41	college education	Cashier
Patient 40	Male	38	Master’s degree	Office clerk
Patient 41	Female	35	Master’s degree	Cashier
Patient 42	Male	36	Bachelor's degree	Retailer

The patients' mental health problems began in March/April 2020. All of the patients also suffered from panic attacks, which caused them to hyperventilate. They also exhibited other common symptoms such as restlessness, sweating, frequent shortness of breath, high blood pressure, body tremors, excessive agitation, and anxiety. All patients were referred to psychological counseling by their family physicians. During the psychological interviews, most of the subjects declared an anxiety prompted by the declaration of the Pandemic in March/April 2020. All subjects declared that they had endured isolation very badly, accompanied by fear of losing their jobs. In addition, lack of contact with some of their family and fear for their health troubled the respondents almost daily after the outbreak of the pandemic. The psychological diagnosis was created from:

Structured Clinical Interview for DSM-SCID- I^46^.

### QEEG

Patients were then subjected to QEEG under clinical standards that represented brainwave amplitudes. This made it possible in the subsequent analysis to compare the neurofeedback training with the patients' baseline and to evaluate its effect. The QEEG examination normally takes about 10–15 min and is divided into two stages of 2–3 min each. The first stage is recording with the eyes closed and the second stage is recording with the eyes open^20^. The tests are performed after obtaining the patient's consent and with his/her complete involvement. Quantitative EEG provides amplitude and power information for specific frequencies and locations on the head. It also indicates the ratios and standard deviations^46^. Quantitative electroencephalography (QEEG) data were collected by a researcher with Board Certification in EEG biofeedback. Taking into account the basic principles of QEEG analysis in an adult (at rest and with eyes open), it is assumed that the lower the frequency of the waves, the lower the amplitude (delta less than 20 mV, theta in adults less than 15 mV, alpha in adults less than 10 mV, SMR, beta1 and beta2 within 4–10 mV). The QEEG data were collected by measuring all waves from central points (Cz, C3, C4), based on the international 10–20 system^46,47^. The curvature of the skull is imaged here in three planes: sagittal, horizontal and coronal^48,49^. The EEG signal was transformed using Cz montage (Cz is the common reference site)^49–51^ and by quantifying with the Elmiko, DigiTrack software (version 14, PL) (ELMIKO, Warsaw, Poland) to examine the central waves asymmetry. The spectrum of a signal is a representation of this signal based on its frequency. Most of the time, the algorithm FFT is used for analysis, with result of the function: f(z) = A(z) + j*F(z). The results of the spectrum analysis in the FFT panel in DigiTrack show amplitudes peak to peak, so as to easily compare results from NF to results from the literature The elimination of artifacts from the EEG recording was performed manually and automatically. The QEEG results of patients with panic attacks are presented below. The study included delta, theta, alpha, SMR, beta1, and beta2 waves at electrodes Fz, Cz, C3, C4, P3, P4, F3, and F4. In this manuscript, we present the results of QEEG diagnostics from the central lane (C3, C4), as EEG Biofeedback therapy was conducted at the same points.

### EEG biofeedback

The test was performed before therapy, after therapy, 6 months after therapy and at follow-up visits. Based on the results provided by the QEEG study, all patients received EEG biofeedback therapy. The main goal was to observe the effectiveness of the therapy in altering brain activity in individuals with panic attacks. All waves, namely Delta, Alpha, Theta, SMR, Beta 1 and Beta 2, from both hemispheres were considered. The C3 and C4 points were used for training. Training sessions were performed for 15 min for each point. Rounds lasted approximately 1–3 min. EEG Biofeedback therapy took place in the central lane in the C3-C4 montage. We started the therapy with the left hemisphere, then moved to the right hemisphere. The frequencies we worked with corresponded to the adult frequencies developed by Sterman Delta 0.5–3 Hz, Theta 4–8 Hz, Alpha 8–12 Hz, SMR 12–15 Hz, Beta 15–20 Hz, Beta2 20–34 Hz. During the training, patients were seated in a chair with a comfortable headrest, slightly elevated legs (relax type chair) and comfortable armrests. During the training, SMR/Theta and SMR/Delta therapeutic protocols with active Beta2 were used to achieve the following relationships in QEEG: Delta > Theta > SMR > Beta1 > Beta2. During EEG Biofeedback training, depending on the round and hemisphere, the patient's attention was focused on a different waveform. With an apparent increase in beta2—the patient would move on to work on beta2, with an increase in theta or delta, to control that particular wave. Everything was done with the cooperation and coordination of the therapist. EEG Biofeedback training is not predictable. They do not follow patterns or templates. The most important thing is a properly performed QEEG diagnostic test, on the basis of which we are able to properly select the therapeutic protocol. The therapist's task is to observe the changes in the EEG and QEEG recordings so that the training sessions are effective and lead to a lasting change in QEEG. QEEG-biofeedback is used to enhance and inhibit the amplitudes of selected waves. The goal we aimed for in all patients was SMR wave enhancement. For Delta, Theta, Beta 1, and Beta 2 waves, inhibition of their amplitudes was desired. In the case of the Alpha wave, the goal was to keep it constant. All training was monitored and controlled for disorderly amplification or inhibition of the waves in question. The thresholds for amplification and inhibition of amplitudes were proportionally changed in response to the changing value of that wave. During the initial training sessions, we also focused on patient education. The patient was taught to observe their own variability in neurophysiological activity in order to influence their own activity and enact changes.

### Statistical analyses

The comparison of the values of quantitative variables in three repeated measurements was performed using the Friedman test. After detecting statistically significant differences, post-hoc analysis (Wilcoxon's paired tests with Bonferroni correction) was performed to identify measurements that are statistically significantly different. A significance level of 0.05 was adopted in the analysis. Thus, all *p* values below 0.05 were interpreted as showing significant relationships. The analysis was performed in the R software, version 4.2.1 (R Core Team (2022). R: A language and environment for statistical computing. R Foundation for Statistical Computing, Vienna, Austria. URL https://www.R-project.org/.).

### Ethics approval

The study was conducted in accordance with the Declaration of Helsinki, and approved by the Ethics Committee of the University of Rzeszow (protocol code 8/12/2021).

### Consent to participate

The studies involving human participants were reviewed and approved by the Ethical Committee of the University of Rzeszow—number 8/12 of permission 8 December 2021. The patients provided written informed consent to participate in this study. No ethical concerns are present.

## Data Availability

The datasets generated during and/or analysed during the current study are available from the corresponding author on reasonable request.
